# Q fever with biliary dilatation following contact with muntjac deer: a case report

**DOI:** 10.3389/fmed.2026.1942956

**Published:** 2026-09-29

**Authors:** Wulong Mao, Yuping Yang, Xinghao Yu, Chenxi Wu, Wei Zhao, Na Huang

**Affiliations:** 1 Department of Respiratory and Critical Care Medicine, The First Affiliated Hospital of Chengdu Medical College, Chengdu, Sichuan, China; 2 Key Laboratory of Geriatric Respiratory Diseases of Sichuan Higher Education Institutes, Chengdu, Sichuan, China; 3 School of Clinical Medicine, The First Affiliated Hospital, Chengdu Medical College, Chengdu, Sichuan, China; 4 Department of Clinical Laboratory, School of Clinical Medicine and the First Affiliated Hospital of Chengdu Medical College, Chengdu, Sichuan, China; 5 School of Laboratory Medicine/Clinical IVD Joint Research Center of Chengdu Medical College-Maccura Biotechnology, Chengdu Medical College, Chengdu, Sichuan, China

**Keywords:** bile duct dilation, *Coxiella burnetii*, muntjac deer, Q fever, zoonotic diseases

## Abstract

Q fever is a zoonosis caused by *Coxiella burnetii*. Classic presentations include atypical pneumonia and hepatitis, but bile duct dilation on imaging is rarely described. We report a case of acute Q fever in an urban resident of Chengdu, Sichuan Province, Southwestern China, who presented with cholestatic liver injury and intra- and extrahepatic bile duct dilation, highlighting an unusual manifestation that mimicked biliary tract disease. A 53-year-old man was admitted with persistent high fever (up to 41.8 °C), headache, chills, and right upper quadrant pain. Laboratory tests showed cholestatic hepatitis (AST 132 U/L, ALT 110 U/L, ALP 221 U/L, GGT 231 U/L), with normal total and direct bilirubin. Magnetic resonance cholangiopancreatography (MRCP) revealed mild intra- and extrahepatic bile duct dilation without obstruction. The patient had no livestock contact but had been exposed to wild muntjac deer from informal sources. Empiric doxycycline combined with piperacillin-tazobactam led to rapid defervescence and symptom improvement. The diagnosis of acute Q fever was supported by metagenomic next-generation sequencing (mNGS) of peripheral blood, in the absence of confirmatory seroconversion. In this case, bile duct dilation without jaundice was a notable manifestation of the infection. For febrile patients with unexplained cholestasis and bile duct dilation, especially those with a history of contact with wild animals, Q fever should be considered in the differential diagnosis.

## Introduction

*Coxiella burnetii*, the causative agent of Q fever, is a strict intracellular Gram-negative bacterium (1). A history of livestock contact or residence in endemic areas has traditionally been considered important for diagnosis (2). However, urbanization and modern transportation have enabled the pathogen to spread to non-endemic, densely populated regions (3). In China, Q fever is not a notifiable disease, and epidemiological data remain scarce (4). Q fever also imposes a substantial public health and economic burden: the 2007–2010 Dutch outbreak, the largest ever recorded, involved more than 4,000 notified human cases and led to extensive livestock losses and economic disruption, with intervention costs at the agricultural level alone reaching approximately €35,000 per disability-adjusted life year (DALY) (3, 5). Chronic Q fever (e.g., endocarditis) also entails long-term morbidity and high healthcare costs (6–8). Consequently, physicians outside traditional endemic areas have limited awareness of this condition, leading to frequent misdiagnosis and delayed treatment. We report a case of acute Q fever in an urban resident who developed fever and cholestatic liver injury after exposure to wild muntjac—an unusual presentation without significant jaundice or biliary obstruction. This case highlights the diagnostic value of early mNGS and, through a literature review of China's Q fever case reports, raises the possibility of a geographic publication bias that warrants attention.

## Case presentation

### Pre-admission medical history

On January 14, 2026, a 53-year-old man was admitted to the Emergency Department of the First Affiliated Hospital of Chengdu Medical College, Chengdu, Sichuan Province, southwestern China, with fever of unknown origin. He reported that the fever had started one week earlier, with a maximum temperature of 41.8 °C, accompanied by headache, chills, profuse sweating, and intermittent sharp pain in the right upper abdomen, back, and both lower legs. Over-the-counter antipyretics were ineffective.

Before transfer to our hospital, he had been evaluated at a local hospital in Qingbaijiang District, Chengdu. Chest and upper abdominal CT revealed scattered chronic pulmonary inflammation, post-cholecystectomy changes, mild intrahepatic and extrahepatic bile duct dilation, slight lymphadenopathy in the hepatogastric space, hepatic hilum, and retroperitoneum, and mild thickening of the left anterior renal fascia.

Local laboratory tests showed mild leukocytosis and abnormal liver function: AST 121.6 U/L, ALT 83.8 U/L, ALP 130.1 U/L, GGT 168.0 U/L; blood cultures were negative. Empiric therapy with ceftriaxone plus oseltamivir normalized his white blood cell count but did not control the fever; instead, inflammatory markers (CRP, SAA, PCT) increased. Because of treatment failure, the patient was transferred to our hospital for further management.

### Post-admission management

On arrival, the patient's temperature was 38 °C. Breath sounds were slightly coarse bilaterally; otherwise physical exam was unremarkable. He was admitted to the respiratory ward. Repeat blood tests, chest CT, and abdominal MRI with magnetic resonance cholangiopancreatography (MRCP; Figure 1) showed similar findings to those from the local hospital. The respiratory team determined that the pulmonary abnormalities and weakly positive influenza A IgM could not explain the persistent high fever (additional laboratory test results are shown in Supplementary Table S1). Considering the abdominal imaging findings and abnormal liver enzymes, they suspected a gastrointestinal or peritoneal origin of infection and consulted the gastroenterology and hepatobiliary surgery departments. Further history taking revealed that the patient had been exposed to wild muntjac deer purchased through informal channels, raising suspicion for a zoonotic infection. Peripheral blood cultures and mNGS were collected on hospital day 2, and a rheumatologic evaluation was also performed (Figure 2A shows a brief timeline of this case).

**Figure 1:**
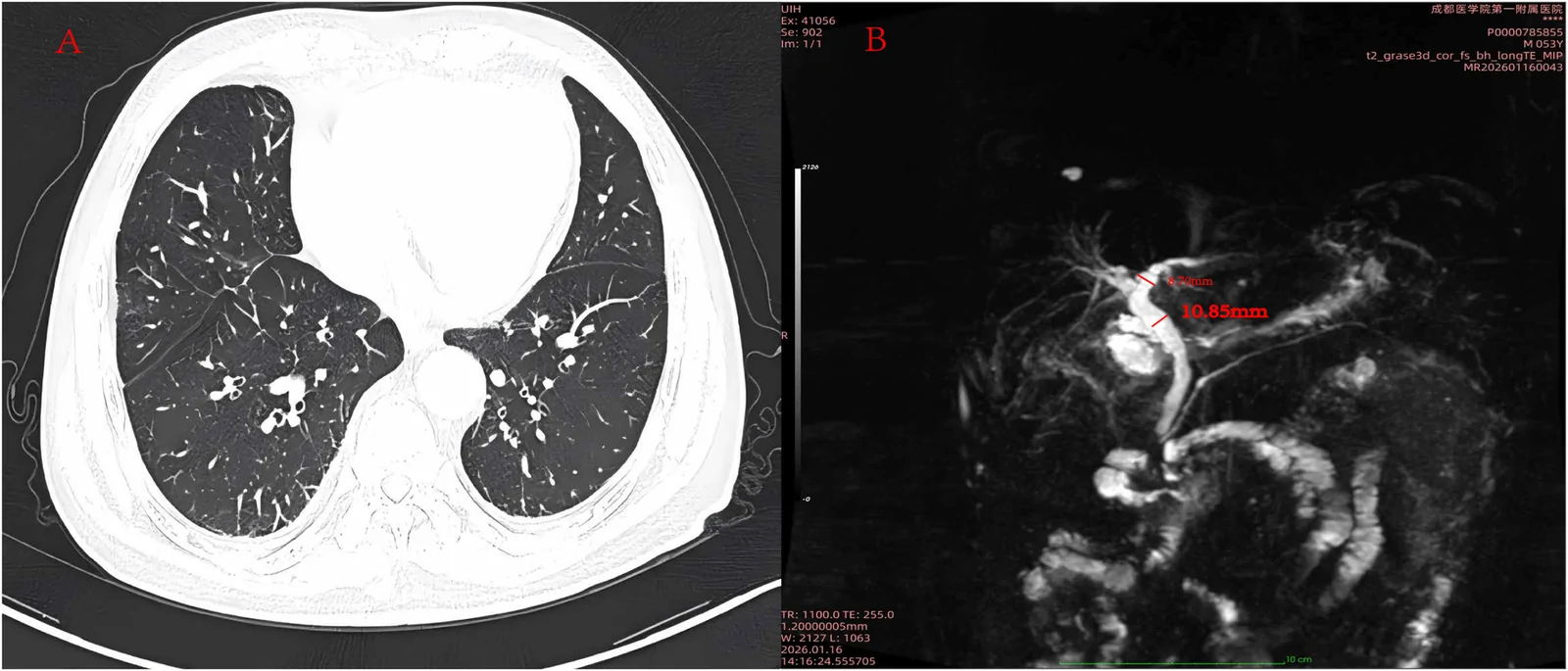
Chest computed tomography (CT) and magnetic resonance cholangiopancreatography (MRCP) findings. **(A)** No significant infectious lesions were identified on the patient's chest CT scan, and the signs of chronic infection did not correlate with the patient's body temperature. Therefore, the medical team largely ruled out a pulmonary origin of infection. **(B)** MRCP showed dilation of the patient's bile ducts, with a maximum diameter of 10.85 mm. The patient had a history of cholecystectomy, but no definite obstructing lesion was identified on MRCP; the cause of the bile duct dilation cannot be fully determined.

**Figure 2:**
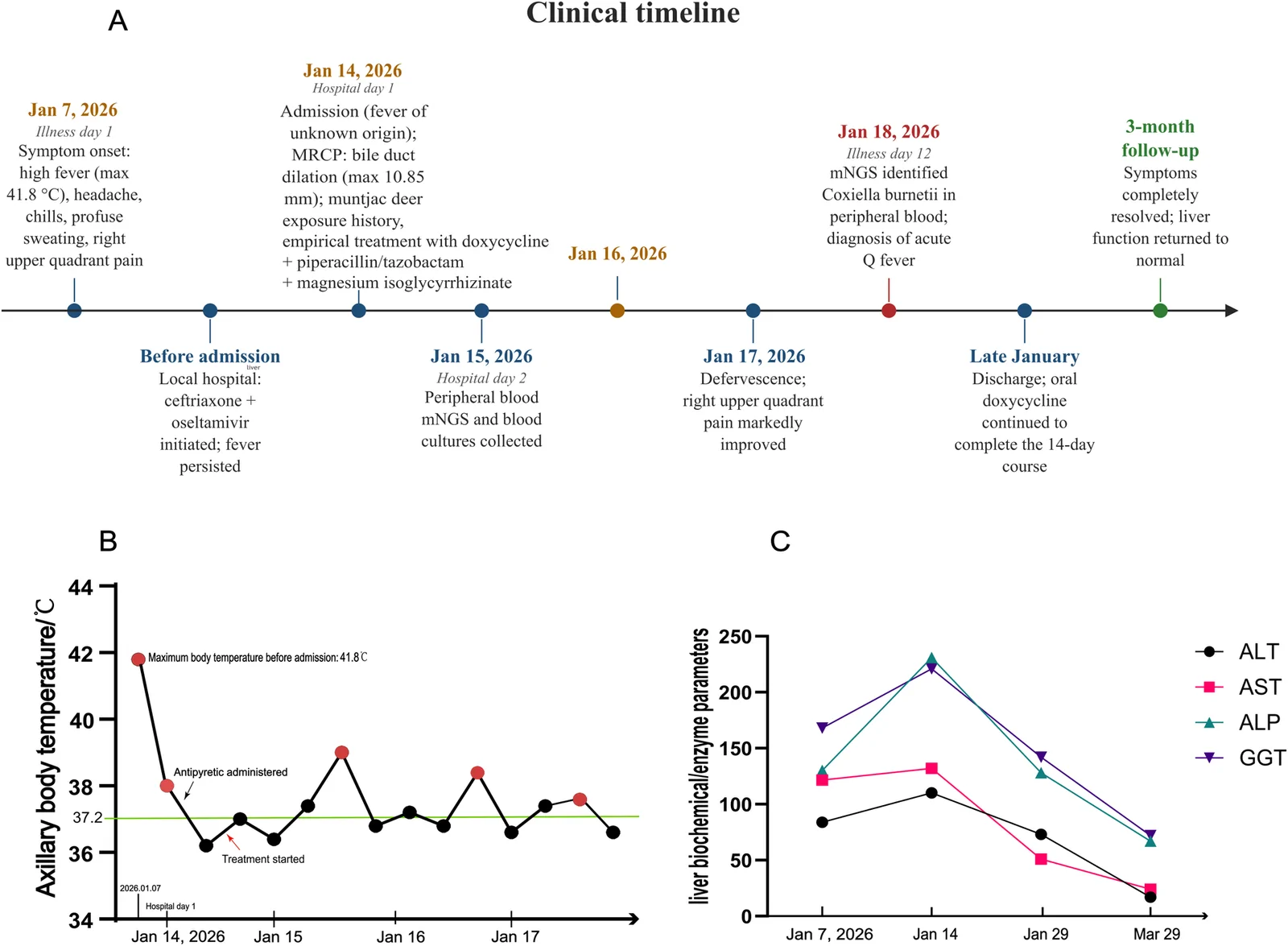
Clinical timeline, axillary temperature, and dynamic changes in liver biochemical parameters. **(A)** A brief timeline of this case. **(B)** After hospitalization, the patient received doxycycline combined with piperacillin-tazobactam (and magnesium isoglycyrrhizinate); the body temperature decreased following this combination therapy. **(C)** The patient's liver biochemical parameters gradually returned to normal during the follow-up period.

### Therapeutic intervention

Piperacillin-tazobactam 450 mg IV q8h + Doxycycline 100 mg PO q12h (double first dose) + Magnesium isoglycyrrhizinate 200 mg IV qd.

### Treatment effect

On the third day of treatment with piperacillin-tazobactam sodium combined with doxycycline, the patient's body temperature decreased significantly (Figure 2B), and the right upper quadrant pain also improved markedly.

On the fourth day, the mNGS report identified *Coxiella burnetii* sequences in the patient's peripheral blood (Table 1), supporting the diagnosis. Consequently, the patient was ultimately diagnosed with Q fever.

**Table 1:** Patient's mNGS test results.

Pathogen	Result	Number of sequences	Relative abundance (%)
Mycoplasma/Chlamydia/Spirochaeta/Rickettsia	*Coxiella burnetii*	384	92.308
Antibiotic resistance gene	not detected		
Virulence gene	not detected		

Sequencing was performed on the MGISEQ-2,000 platform (Wuhan MGI Tech Co., Ltd., China).

### Follow-up and outcomes

After discharge, the patient continued oral doxycycline therapy to complete the full 14-day course. During this period, his symptoms completely resolved without any sequelae. At the 3-month follow-up, his liver function and other indicators gradually returned to normal (Figure 2C).

## Discussion

### Biliary dilation as an unusual manifestation of Q fever in this case

A 53-year-old man presented with persistent high fever, headache, and chills. Outside hospital imaging showed chronic lung inflammation, biliary abnormalities, and mild liver dysfunction. Empiric therapy with a cephalosporin plus oseltamivir failed to control his fever. He lived in an urban area with no livestock exposure but had contact with wild animals from informal sources shortly before symptom onset.

Acute Q fever typically manifests as self-limited fever, atypical pneumonia, or granulomatous hepatitis (9). Liver function abnormalities are common but usually mild to moderate, with elevations of transaminases (AST, ALT) and GGT. Bile duct dilation on imaging has rarely been reported, and major reviews do not list it as a common radiographic finding (6, 7). Several previous case reports have described cholestatic jaundice and hepatitis in patients with Q fever (10, 11). However, in our case, no significant jaundice was observed, only mild hepatic impairment and biliary dilation, as well as elevated GGT and ALP levels with normal total and direct bilirubin (Supplementary Table S1). The absence of jaundice in the setting of biliary dilation is consistent with an inflammatory component rather than mechanical obstruction, although the mechanisms remain incompletely understood. Systemic cytokines, particularly IL-6 and TNF-α, may downregulate bile acid transporter expression and contribute to cholestatic liver injury without structural obstruction (12); these mechanisms are biologically plausible, but they were not directly demonstrated in this patient. Although prior biliary tract examination results were unavailable to rule out congenital biliary dilation, the patient recalled that previous tests revealed no evidence of bile duct dilation or biliary stones. The patient's biliary imaging findings were atypical for Q fever, and this atypical clinical presentation led to a delayed initial diagnosis, which was confirmed only after obtaining metagenomic sequencing (mNGS) results. Clinically, in febrile patients with cholestatic liver injury or bile duct dilation, Q fever should be considered after excluding primary biliary diseases—especially when there is a history of animal contact, even in the absence of typical pneumonia or hepatitis.

### mNGS plays a crucial role in the diagnosis of fever of unknown origin

The diagnosis of *Coxiella burnetii* infection was supported by peripheral blood mNGS; confirmatory seroconversion was not documented. mNGS testing was performed by the Clinical Laboratory of the First Affiliated Hospital of Chengdu Medical College, an ISO 15189 (CNAS)-accredited laboratory, using the MGISEQ-2000 platform (Wuhan MGI Tech Co., Ltd., China). Serology, while useful, has a window period and cannot provide early pathogen identification (13). mNGS overcomes this limitation and is valuable in critical, emerging, rare, or mixed infections (14, 15). A 2024 Chinese consensus recommended prioritizing mNGS for patients with negative conventional tests but high suspicion of atypical pathogens (16). This case supports early mNGS in patients with progressive fever of unknown origin, as it shortens diagnostic time and guides treatment.

In our patient, symptoms began on January 7, 2026; blood was drawn for mNGS on January 15 (day 8 of illness). The result returned on January 19 (day 12 of illness). At the time of writing, serological tests for specific pathogens (apart from a weakly positive influenza A IgM) remained negative. Thus, had we relied solely on serology, the diagnosis would have been missed or delayed by at least another week. mNGS provided a definitive answer on day 12, allowing a confident diagnosis.

While mNGS is more expensive than serology, the cost should be weighed against the consequences of delayed or missed diagnosis—including unnecessary invasive procedures (e.g., endoscopic retrograde cholangiopancreatography for suspected biliary obstruction) and progression to chronic Q fever (17). Therefore, we suggest that in patients with progressive fever of unknown origin and atypical features (such as bile duct dilation), early mNGS may be cost-effective even in resource-limited settings.

### Muntjac deer and wildlife exposure: an emerging risk for Q fever

Although Q fever infection has traditionally been associated with inhalation of aerosols generated by infected livestock (18), some studies have demonstrated that oral exposure can also lead to infection, with oral transmission clearly validated in mouse models (19). Additionally, raw milk contaminated with *Coxiella burnetii* is recognized as a significant potential source of infection; for example, the Netherlands reported over 4,000 cases of Q fever between 2007 and 2010, most of which were linked to goat farms—marking the largest recorded outbreak of Q fever (5, 20, 21).

Our case suggests that exposure to wild muntjac deer through informal channels—whether by handling, dressing, or consumption—may represent a transmission route, even without direct contact with farm animals. Currently, relevant studies from Portugal have detected antibodies against *Coxiella burnetii* in wild deer species such as red deer and wild boar; however, publicly available literature on serological surveys or PCR testing for Q fever in muntjac deer (*Muntiacus* spp.) in China remains scarce, with few reports of infections caused by these animals (22). A 2010 report from a small village in Taiwan described a Q fever outbreak presumably associated with exposure to Formosan barking deer (*Muntiacus reevesi*), a muntjac species (23).

It must be noted that there are historical precedents of major public health events triggered by wildlife consumption—the 2003 SARS outbreak linked to palm civets being a typical example (24, 25). Although pathogens associated with outbreaks such as SARS differ significantly from those reported in this case, rare pathogen infections associated with exposure to wild animals warrant sufficient attention. Despite explicit legal prohibitions, illegal wildlife trade and consumption still persist in certain regions of China. While the source of infection could not be confirmed, this case raises the possibility that *Coxiella* may circulate among wild deer populations in Sichuan and surrounding areas; this hypothesis requires validation through serological and molecular surveillance. Clinicians should routinely inquire whether patients with fever who test negative on routine examinations have had exposure to wild game (including handling, dressing, or consumption), rather than focusing solely on contact with domestic livestock.

### Regional distribution of China Q fever case reports: genuine variation or publication bias?

As mentioned earlier, because Q fever is not systematically reported or tested for in China, relevant epidemiological data are severely scarce. To gain a preliminary understanding of the reporting situation of this disease within China, we searched PubMed using the following strategy: (“Q Fever”[Mesh] OR Coxiella burnetii) AND (China[MeSH Terms] OR China[Affiliation] OR China[tiab] OR Taiwan[MeSH Terms] OR Taiwan[Affiliation] OR Taiwan[tiab]) AND (“Case Reports”[pt]) AND 2000/01/01:2025/12/31[dp]. The search retrieved 57 publications. After full-text screening, 56 studies were included; one study was excluded because the reported cases were from Thailand. The included studies reported 74 individual cases. Each publication was classified by the region of the reported case(s); when the number of cases was not explicitly stated, it was counted as one case (the full list of included publications is provided in Supplementary Table S2). Because the search was restricted to publications indexed under the “Case Reports” publication type, outbreak reports and other study types not indexed under this heading (e.g., the 2010 Taiwanese muntjac-deer-associated outbreak (23) were not included in this inventory. Guangdong, Beijing, Zhejiang, and Taiwan (China) reported more cases than other regions (Figure 3). Most of these reports appeared after 2020. This distribution differs from the traditional view of Q fever as a “pastoral disease”. Several factors may explain this pattern. First, economically developed regions have better access to advanced tests such as mNGS, resulting in higher detection rates. Second, regions with more research resources are more likely to publish in international journals, while cases from western pastoral areas may be more often reported in Chinese-language journals not indexed in PubMed. Third, the COVID-19 pandemic may have promoted the use of molecular diagnostics, further increasing detection. Therefore, the apparent regional distribution based on published reports raises the possibility of a geographic publication bias that warrants attention.

**Figure 3:**
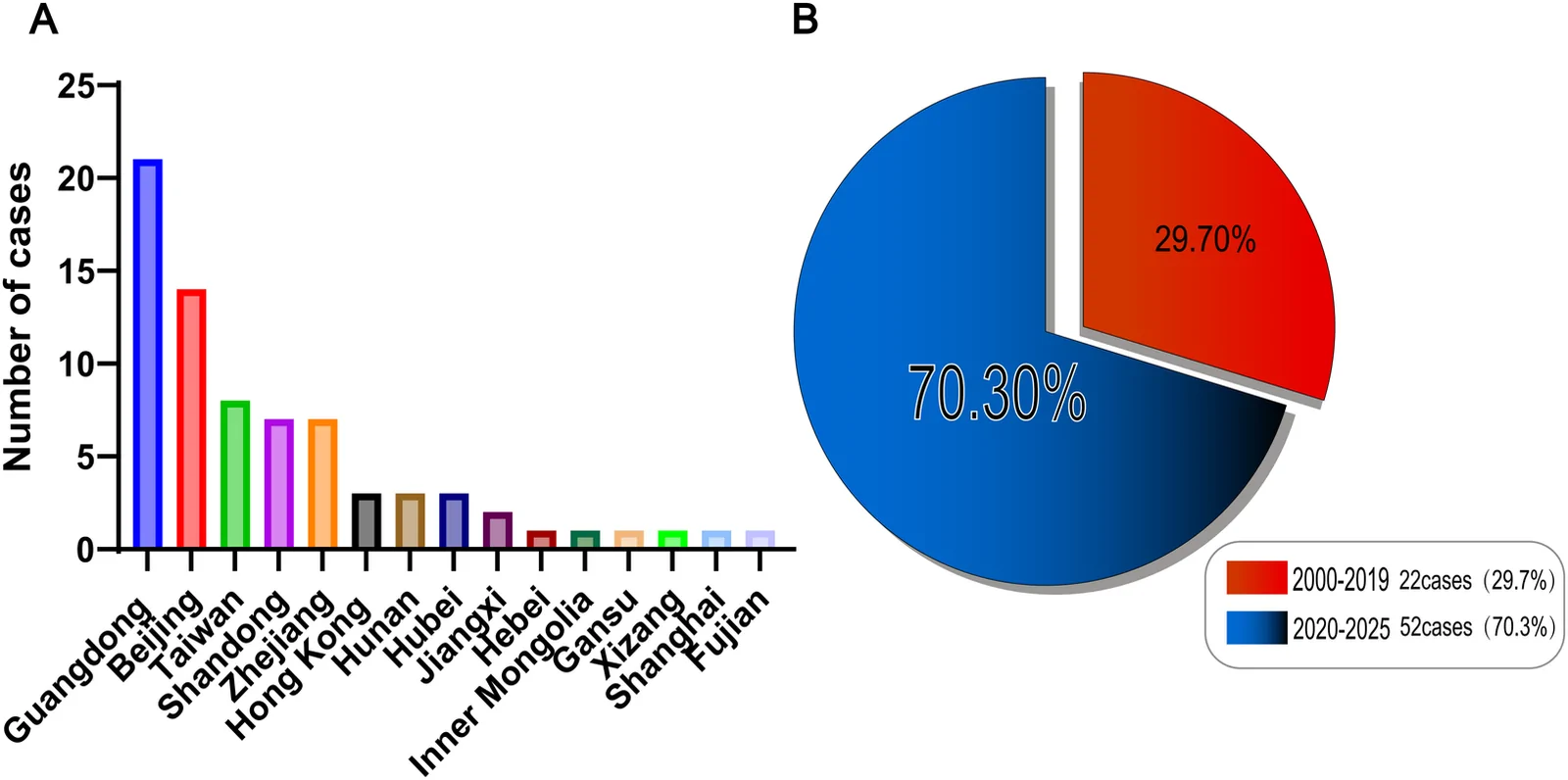
Geographical and temporal distribution of Chinese Q fever case reports (2000–2025). **(A)** Regional distribution of 56 Q fever case report publications (74 individual cases) from China retrieved from PubMed between 2000 and 2025; economically developed urbanized areas such as Guangzhou and Beijing reported higher case numbers compared with other regions; the complete list of included publications is provided in Supplementary Table S2. **(B)** The number of reported Q fever cases in China increased significantly between 2020 and 2025.

### The importance of Q fever awareness among medical professionals

Q fever remains a neglected zoonotic disease in many countries, and a substantial proportion of medical professionals lack adequate knowledge about it. A study conducted in Brazil assessing the level of knowledge among healthcare professionals regarding Q fever found that 92.9% of physicians lacked understanding of the epidemiological characteristics, clinical manifestations, diagnostic methods, and preventive measures related to Q fever (26). Such knowledge gaps directly contribute to underdiagnosis and delayed treatment, which can lead to severe acute disease, progression to chronic Q fever, and, in some cases, death.

Improving knowledge of Q fever among physicians is therefore essential for timely diagnosis and appropriate management. Greater dissemination of information through medical education, clinical guidelines, and continuing professional training should be encouraged, and clinicians should be reminded to take a detailed animal-exposure history in febrile patients. Because Q fever links human, animal, and environmental health, a One Health approach—with collaboration among medical, veterinary, and public health professionals—has been advocated for the control of Q fever and other neglected zoonoses (27).

### Limitations

The main limitations of this case are the lack of paired serology and the single-case nature. Diagnosis relied on mNGS; we did not document phase II seroconversion because the patient declined follow-up blood sampling after symptoms resolved. Routine serology (except for weak influenza A IgM) remained negative throughout the illness. We could not test the suspected source—muntjac deer obtained informally—nor verify the exposure history beyond the patient's recall. Pre-illness biliary imaging was unavailable, so pre-existing or congenital dilation cannot be excluded. Finally, the 3-month follow-up is too short to fully exclude chronic Q fever, and our PubMed-based literature search, which relied on a single database and limited search terms, may not capture all Chinese reports. In addition, the proposed mechanisms for biliary dilation were not directly demonstrated in this patient (no liver biopsy was performed).

## Conclusions

Clinicians should consider Q fever in febrile patients with unexplained cholestasis and bile duct dilation, even without jaundice, obstruction, or livestock exposure. In this patient, mild bile duct dilation occurred in association with acute Q fever; whether it reflected inflammation rather than a surgical lesion could not be definitively established, and infectious causes, including Q fever, should be considered in the differential diagnosis before invasive procedures are undertaken when imaging does not clearly indicate mechanical obstruction. A history of wildlife exposure, particularly contact with muntjac deer (including handling, dressing, or consumption), should be actively sought, although the source of infection could not be confirmed in this case. In this patient, empirical doxycycline—consistent with established indications for suspected Q fever—and metagenomic next-generation sequencing were valuable when serology remained in the window period. The geographic distribution of China's Q fever case reports raises the possibility of a geographic publication bias that warrants attention.

## Data Availability

The original contributions presented in the study are included in the article/Supplementary Material, further inquiries can be directed to the corresponding authors.
