# Supplementary material for: Q fever with biliary dilatation following contact with muntjac deer: a case report
Source: Front Med (Lausanne). 2026 Sep 29;13:1942956. doi: 10.3389/fmed.2026.1942956 (PMC13657949; doi:10.3389/fmed.2026.1942956)
Supplement: Supplementary file 1 [file Datasheet1.pdf]

Supplementary Table 1. Part of laboratory test results after the patient's admission

Laboratory Test Items	unit	result	Reference range
White blood cell count	10^9/L	3.43	3.5-9.5
Red blood cell count	10^{12}/L	3.25	4.3-5.8
Hemoglobin	g/L	107	130-175
Platelet count	10^9/L	112	125-350
Monocyte percentage	%	2.8	3-10
Erythrocyte sedimentation rate	mm/h	54	0-15
Procalcitonin	ng/ml	0.846	<0.046
Alanine aminotransferase	U/L	110	9-50
Aspartate aminotransferase	U/L	132	15-40
Gamma-glutamyl transpeptidase	U/L	231	11-49
Alkaline phosphatase	U/L	221	45-125
Lactate dehydrogenase	U/L	504	120-250
α-hydroxybutyrate Dehydrogenase	U/L	346	72-182
Total bilirubin	μmol/L	10.3	≤23.0
Direct bilirubin	μmol/L	4.8	0-8
Indirect bilirubin	μmol/L	5.5	NA
Total bile acid	μmol/L	16.6	0-10

Supplementary Table 2. PubMed (2000 – 2025): Q fever case statistics

literature	Caselocation	caseload	Reportingyear/Onsetyear
DOI: 10.1016/j.kint.2024.12.023	Beijing	1	2025
DOI: 10.1186/s12879-022-07938-7	Shandong	1	2023
DOI: 10.1371/journal.pntd.0010364	Guangdong	1	2014
	Guangdong	2	2019
	Guangdong	4	2020
	Guangdong	7	2021
DOI: 10.1016/j.bsheal.2024.11.003	Jiangxi	1	2024
DOI: 10.3855/JIDC.18314	Hunan	1	2024
DOI: 10.1016/J.DiagMicrobio.2024.116594	Shandong	1	2025
DOI: 10.1111/os.13600	Fujian	1	2022
PMID: 24767204.	Beijing	4	2014
DOI: 10.1016/j.jgar.2025.09.015	Zhejiang	1	2022
DOI: 10.2147/IDR.S566918	Zhejiang	1	2023
	Zhejiang	1	2025
DOI: 10.1186/S12866-021-02323-X	Hubei	1	2021
DOI: 10.1186/s12879-022-07309-2	Beijing	1	2020
DOI: 10.1016/J.DiagMicrobio.2024.116434	Guangdong	1	2024
DOI: 10.1186/S12879-025-10699-8	Zhejiang	1	2025

literature	Caselocation	caseload	Reportingyear/Onsetyear
DOI: 10.3760/CMA.J.CN112147-20240113-00028	Beijing	1	2024
DOI: 10.3389/fmed.2025.1636778	Hebei	1	2025
DOI: 10.1177/03000605211025398	Zhejiang	1	2021
DOI: 10.3389/fcimb.2024.1323054	Shandong	1	2024
DOI: 10.2147/IDR.S495710	Beijing	1	2024
DOI: 10.12968/HMED.2024.0604	Beijing	1	2021
DOI: 10.1097/MD.0000000000041407	Inner Mongolia	1	2023
DOI: 10.2147/IDR.S405697	Hubei	1	2022
DOI: 10.3389/FMED.2025.1618670	Gansu	1	2023
PMID: 20124575	Hong Kong	1	2008
DOI: 10.3760/CMA.J.CN112147-20250326-00165	Tibet	1	2024
DOI: 10.3389/FMED.2025.1584216	Guangdong	1	2022
DOI: 10.1016/J.DiagMicrobio.2025.117097	Beijing	1	2024
DOI: 10.1186/s12879-025-12102-y	Guangdong	1	2025
DOI: 10.3892/ETM.2024.12764	Beijing	1	2024
DOI: 10.1186/s12879-025-10589-z	Hubei	1	2025
DOI: 10.3389/fmed.2022.855020	Hunan	1	2022
DOI: 10.1016/J.ijid.2026.108467	Hunan	1	2025
DOI: 10.3389/FMED.2025.1656891	Zhejiang	1	2023
DOI: 10.1007/S10096-025-05297-0	Guangdong	1	2021
DOI: 10.1016/j.jvscit.2025.101949	Guangdong	1	2022
DOI: 10.2147/IDR.S485083	Beijing	1	2023
DOI: 10.1097/MD.0000000000046734	Shandong	1	2022
DOI: 10.2147/IDR.S361969	Shandong	1	2021
DOI：10.1016/J.IMJ.2025.100212	Jiangxi	1	2024
DOI: 10.12809/hkmj208622	Hong Kong	1	2021
DOI: 10.3389/fmed.2026.1701814	Shandong	1	2026
DOI: 10.3389/fmed.2025.1626115	Zhejiang	1	2025
DOI: 10.1016/S1607-551X(09)70161-5	Taiwan	1	2005
DOI: 10.1016/J.BJID.2014.11.001.	Shanghai	1	2015
PMID: 16996015	Beijing	1	2004
DOI: 10.1007/S12350-015-0080-0	Guangdong	1	2015
DOI: 10.3760/cma.j.cn112138-20200407-00350	Shandong	1	2019
DOI: 10.1111/ijlh.70039.	Guangdong	1	2025
PMID: 25124913	Beijing	1	2011
DOI: 10.1016/S0929-6646（08）60207-7	Taiwan	1	2005
DOI: 10.1097/01.RLU.0000099816.32114.9c	Taiwan	1	2003
DOI: 10.1093/CID/CIS652	Hong Kong	1	2012
PMID: 15181492	Taiwan	1	2009
PMID: 22116335	Taiwan	2	2004
DOI: 10.1186/1471-2334-10-193	Taiwan	1	2010
DOI: 10.1186/s12879-014-0651-8	Taiwan	1	2014
